# Supplementary material for: COZOID: contact zone identifier for visual analysis of protein-protein interactions
Source: BMC Bioinformatics. 2018 Apr 6;19:125. doi: 10.1186/s12859-018-2113-6 (PMC5889581; doi:10.1186/s12859-018-2113-6)
Supplement: Supplementary file 2 — Software build. Executable binary file of the software tool. (ZIP 118,784 kb) [file 12859_2018_2113_MOESM2_ESM.zip › cozoid/java/docs/beansbinding-1.2.1-doc.zip!/org/jdesktop/beansbinding/Property.html]

Property (Beans Binding)


---


|  |  |  |
| --- | --- | --- |
| **Overview** \| **Package** \| **Class** \| **Use** \| **Tree** \| **Deprecated** \| **Index** \| **Help** | |  |
| **PREV CLASS**   **NEXT CLASS** | **FRAMES**    **NO FRAMES**     **All Classes** |  |
| SUMMARY: NESTED \| FIELD \| CONSTR \| METHOD | DETAIL: FIELD \| CONSTR \| METHOD |  |


---


## org.jdesktop.beansbinding Class Property<S,V>

```
java.lang.Object
  org.jdesktop.beansbinding.Property<S,V>
```

**Type Parameters:**: `S` - the type of source object that this `Property` operates on: `V` - the type of value that this `Property` represents

**Direct Known Subclasses:**: ObjectProperty, PropertyHelper

---

``` public abstract class Property<S,V> extends java.lang.Object ```

`Property` defines a uniform way to access the value of a property.
A typical `Property` implemention allows you to create an immutable
representation of a way to derive some property from a source object.
As such, all methods of this class take a source object as an argument.

A `Property` implementation may, however, be designed such that the
`Property` itself is a mutable thing that stores a property value.
In such a case, the `Property` implementation may ignore the source
object. `Property` implementations should clearly document their
behavior in this regard.

You can listen for changes in the state of a `Property` by
registering `PropertyStateListeners` on the `Property`.

---

**Constructor Summary**

`Property()`


| **Method Summary** | |
| --- | --- |
| `abstract  void` | `addPropertyStateListener(S source, PropertyStateListener listener)`             Adds a `PropertyStateListener` to be notified when the state of the `Property` changes with respect to the given source. |
| `abstract  PropertyStateListener[]` | `getPropertyStateListeners(S source)`             Returns an arry containing the listeners registered for the given source. |
| `abstract  V` | `getValue(S source)`             Returns the value of this `Property` for the given source. |
| `abstract  java.lang.Class<? extends V>` | `getWriteType(S source)`             Returns the type of object that is suitable for setting as the value of this `Property` by calls to `setValue`. |
| `abstract  boolean` | `isReadable(S source)`             Returns whether or not the `Property` is readable for the given source. |
| `abstract  boolean` | `isWriteable(S source)`             Returns whether or not the `Property` is writeable for the given source. |
| `abstract  void` | `removePropertyStateListener(S source, PropertyStateListener listener)`             Removes a `PropertyStateListener` for the given source. |
| `abstract  void` | `setValue(S source, V value)`             Sets the value of this `Property` for the given source. |

**Methods inherited from class java.lang.Object**

`clone, equals, finalize, getClass, hashCode, notify, notifyAll, toString, wait, wait, wait`

**Constructor Detail**

### Property

```
public Property()
```


**Method Detail**

### getWriteType

```
public abstract java.lang.Class<? extends V> getWriteType(S source)
```

:   Returns the type of object that is suitable for setting as the value
    of this `Property` by calls to `setValue`.

    :   **Parameters:**: `source` - the source object on which to operate **Returns:**: the type of object suitable for setting as the value **Throws:**: `java.lang.UnsupportedOperationException` - if the `Property` is not writeable for the given source **See Also:**: `setValue(S, V)`, `isWriteable(S)`

---


### getValue

```
public abstract V getValue(S source)
```

:   Returns the value of this `Property` for the given source.

    :   **Parameters:**: `source` - the source object on which to operate **Returns:**: the value of this `Property` for the given source **Throws:**: `java.lang.UnsupportedOperationException` - if the `Property` is not readable for the given source **See Also:**: `isReadable(S)`

---


### setValue

```
public abstract void setValue(S source,
                              V value)
```

:   Sets the value of this `Property` for the given source.

    :   **Parameters:**: `source` - the source object on which to operate: `value` - the new value for the `Property` **Throws:**: `java.lang.UnsupportedOperationException` - if the `Property` is not writeable for the given source **See Also:**: `isWriteable(S)`, `getWriteType(S)`

---


### isReadable

```
public abstract boolean isReadable(S source)
```

:   Returns whether or not the `Property` is readable for the given source.

    :   **Parameters:**: `source` - the source object on which to operate **Returns:**: whether or not the `Property` is readable for the given source. **See Also:**: `isWriteable(S)`

---


### isWriteable

```
public abstract boolean isWriteable(S source)
```

:   Returns whether or not the `Property` is writeable for the given source.

    :   **Parameters:**: `source` - the source object on which to operate **Returns:**: whether or not the `Property` is writeable for the given source. **See Also:**: `isReadable(S)`

---


### addPropertyStateListener

```
public abstract void addPropertyStateListener(S source,
                                              PropertyStateListener listener)
```

:   Adds a `PropertyStateListener` to be notified when the state of the
    `Property` changes with respect to the given source. Does nothing if
    the listener is `null`. If a listener is added more than once,
    notifications are sent to that listener once for every time that it has
    been added. The ordering of listener notification is unspecified.

    :   **Parameters:**: `source` - the source object on which to operate: `listener` - the listener to be notified

---


### removePropertyStateListener

```
public abstract void removePropertyStateListener(S source,
                                                 PropertyStateListener listener)
```

:   Removes a `PropertyStateListener` for the given source. Does
    nothing if the listener is `null` or is not one of those registered
    for this source object. If the listener being removed was registered more
    than once, only one occurrence of the listener is removed from the list of
    listeners. The ordering of listener notification is unspecified.

    :   **Parameters:**: `source` - the source object on which to operate: `listener` - the listener to be removed **See Also:**: `addPropertyStateListener(S, org.jdesktop.beansbinding.PropertyStateListener)`

---


### getPropertyStateListeners

```
public abstract PropertyStateListener[] getPropertyStateListeners(S source)
```

:   Returns an arry containing the listeners registered for the given source.
    Order is undefined. Returns an empty array if there are no listeners.

    :   **Parameters:**: `source` - the source object on which to operate **Returns:**: the set of listeners registered for the given source **See Also:**: `addPropertyStateListener(S, org.jdesktop.beansbinding.PropertyStateListener)`


---


|  |  |  |
| --- | --- | --- |
| **Overview** \| **Package** \| **Class** \| **Use** \| **Tree** \| **Deprecated** \| **Index** \| **Help** | |  |
| **PREV CLASS**   **NEXT CLASS** | **FRAMES**    **NO FRAMES**     **All Classes** |  |
| SUMMARY: NESTED \| FIELD \| CONSTR \| METHOD | DETAIL: FIELD \| CONSTR \| METHOD |  |


---
